# Healthcare providers’ acceptance of telemedicine and preference of modalities during COVID-19 pandemics in a low-resource setting: An extended UTAUT model

**DOI:** 10.1371/journal.pone.0250220

**Published:** 2021-04-22

**Authors:** Kirubel Biruk Shiferaw, Shegaw Anagaw Mengiste, Monika Knudsen Gullslett, Atinkut Alamirrew Zeleke, Binyam Tilahun, Tsion Tebeje, Robel Wondimu, Surafel Desalegn, Eden Abetu Mehari

**Affiliations:** 1 Health Informatics Department, College of Medicine and Health Science, Debre Markos University, Debre Markos, Ethiopia; 2 School of Business, Institute of Business, History & Social Sciences, University of South-Eastern Norway, Notodden, Norway; 3 Faculty of Health & Social Sciences, Science Center Health & Technology, University of South-Eastern Norway, Drammen, Norway; 4 Medical Informatics, Institute for Community Medicine, University Medicine Greifswald, Greifswald, Germany; 5 Department of Health Informatics, Institute of Public Health, College of Medicine and Health Science, University of Gondar, Gondar, Ethiopia; 6 Department of Internal Medicine, Borre General Hospital, Borre, Ethiopia; 7 Department of Emergency Medicine, School of Medicine, College of Medicine and Health Science, University of Gondar, Gondar, Ethiopia; 8 Department of Clinical Pharmacy, School of Pharmacy, College of Medicine and Health Science, University of Gondar, Gondar, Ethiopia; Catholic University of the North, CHILE

## Abstract

**Background:**

In almost all lower and lower middle-income countries, the healthcare system is structured in the customary model of in-person or face to face model of care. With the current global COVID-19 pandemics, the usual health care service has been significantly altered in many aspects. Given the fragile health system and high number of immunocompromised populations in lower and lower-middle income countries, the economic impacts of COVID-19 are anticipated to be worse. In such scenarios, technological solutions like, Telemedicine which is defined as the delivery of healthcare service remotely using telecommunication technologies for exchange of medical information, diagnosis, consultation and treatment is critical. The aim of this study was to assess healthcare providers’ acceptance and preferred modality of telemedicine and factors thereof among health professionals working in Ethiopia.

**Methods:**

A multi-centric online survey was conducted via social media platforms such as telegram channels, Facebook groups/pages and email during Jul 1- Sep 21, 2020. The questionnaire was adopted from previously validated model in low income setting. Internal consistency of items was assessed using Cronbach alpha (α), composite reliability (CR) and average variance extracted (AVE) to evaluate both discriminant and convergent validity of constructs. The extent of relationship among variables were evaluated by Structural equation modeling (SEM) using SPSS Amos version 23.

**Results:**

From the expected 423 responses, 319 (75.4%) participants responded to the survey questionnaire during the data collection period. The majority of participants were male (78.1%), age <30 (76.8%) and had less than five years of work experience (78.1%). The structural model result confirmed the hypothesis “self-efficacy has a significant positive effect on effort expectancy” with a standardized coefficient estimate (β) of 0.76 and p-value <0.001. The result also indicated that self-efficacy, effort expectancy, performance expectancy, facilitating conditions and social influence have a significant direct effect on user’s attitude toward using telemedicine. User’s behavioral intention to use telemedicine was also influenced by effort expectancy and attitude. The model also ruled out that performance expectancy, facilitating conditions and social influence does not directly influence user’s intention to use telemedicine. The squared multiple correlations (r^2^) value indicated that 57.1% of the variance in attitude toward using telemedicine and 63.6% of the variance in behavioral intention to use telemedicine is explained by the current structural model.

**Conclusion:**

This study found that effort expectancy and attitude were significantly predictors of healthcare professionals’ acceptance of telemedicine. Attitude toward using telemedicine systems was also highly influenced by performance expectancy, self-efficacy and facilitating conditions. effort expectancy and attitude were also significant mediators in predicting users’ acceptance of telemedicine. In addition, mHealth approach was the most preferred modality of telemedicine and this opens an opportunity to integrate telemedicine systems in the health system during and post pandemic health services in low-income countries.

## Background

The world health organization (WHO) declared the outbreak of novel corona virus (COVID-19) on January 30^th^ 2020 as a public health emergency in china and later as a global pandemic on march 11^th^ 2020 [1]. Due to the virus’s contagious and complex nature of transmission, nearly 68 million peoples were infected and almost 1.6 million people were dead globally during the study period of this study [2]. The WHO emergency committee have suggested early detection, isolation, contact tracing and treatment in response to the pandemics to interject the exponential transmission of the virus. As a result of safety measures like transportation restriction (international and national level), school closing and quarantine (Stay home) measures, there is a clear potential for prolonged economic crisis or recession as there is no cure for the disease yet [3]. Given the fragile health system and high number of immunocompromised papulations in lower and lower-middle income countries, the economic impacts of COVID-19 are anticipated to be worse [4]. Until drugs/vaccines are widely available, safety and preventive measures like physical distancing (staying at home), hygiene, disinfecting and case isolation are the existing protective measures. On the other hand, the advancement of technology abruptly changed the business process of almost all sectors in the world and the health sector is one of the major areas where technological advancement improves the lives of millions. The developed nations are highly equipped with the infrastructural and skilled labor force when compared to the rest of the world which enable them to stay resilient and protective against their national threats like COVID-19. In almost all lower and lower middle income countries, the healthcare system is structured in the customary model of in-person or face to face model of care and resulted from the pandemics, the usual health care service has been significantly altered in many aspects [5]. People with underlying conditions like chronic disease or other infectious comorbidities are strangled between making a difficult choice between contracting COVID-19 in expense of seeking healthcare service (clinical visit) and staying home and take care of themselves as much as possible. In the time of global crisis like COVID-19, an advanced approach of addressing healthcare service is priceless. Considering the serious challenge of physicians/patient contact in person due to such inevitable global crises, applying tech-based approaches such as telemedicine which enables to carry on the regular healthcare service maintaining physical inaccessibility is crucial.

Telemedicine is defined as the delivery of healthcare service using telecommunication technologies for exchange of medical information, diagnosis, consultation and treatment where physical distance is a critical issue [6]. In such difficult times, evidences suggest using telemedicine is feasible, acceptable, and effective in improving in health care outcomes and can maintain long distance healthcare service. The most common modalities of telemedicine include real-time technology, store-and-forward technology, remote monitoring and M-health approaches [7–9].

Given the infrastructural and skilled human resource limitations in low-income settings, assessing healthcare provider’s acceptance and preference of telemedicine modalities during the deadly pandemics is an important information to tailor telemedicine modalities to a specific context. The aim of this study was to assess healthcare providers’ acceptance and preferred modality of telemedicine and factors thereof among health professionals working in COVID-19 operation sections in Ethiopia. The Model used to explain the variance in acceptance of telemedicine is based on the Extended version of Unified Theory of Acceptance and Use of Technology (UTAUT) model [10].

### Theoretical background

**Fig 1:**
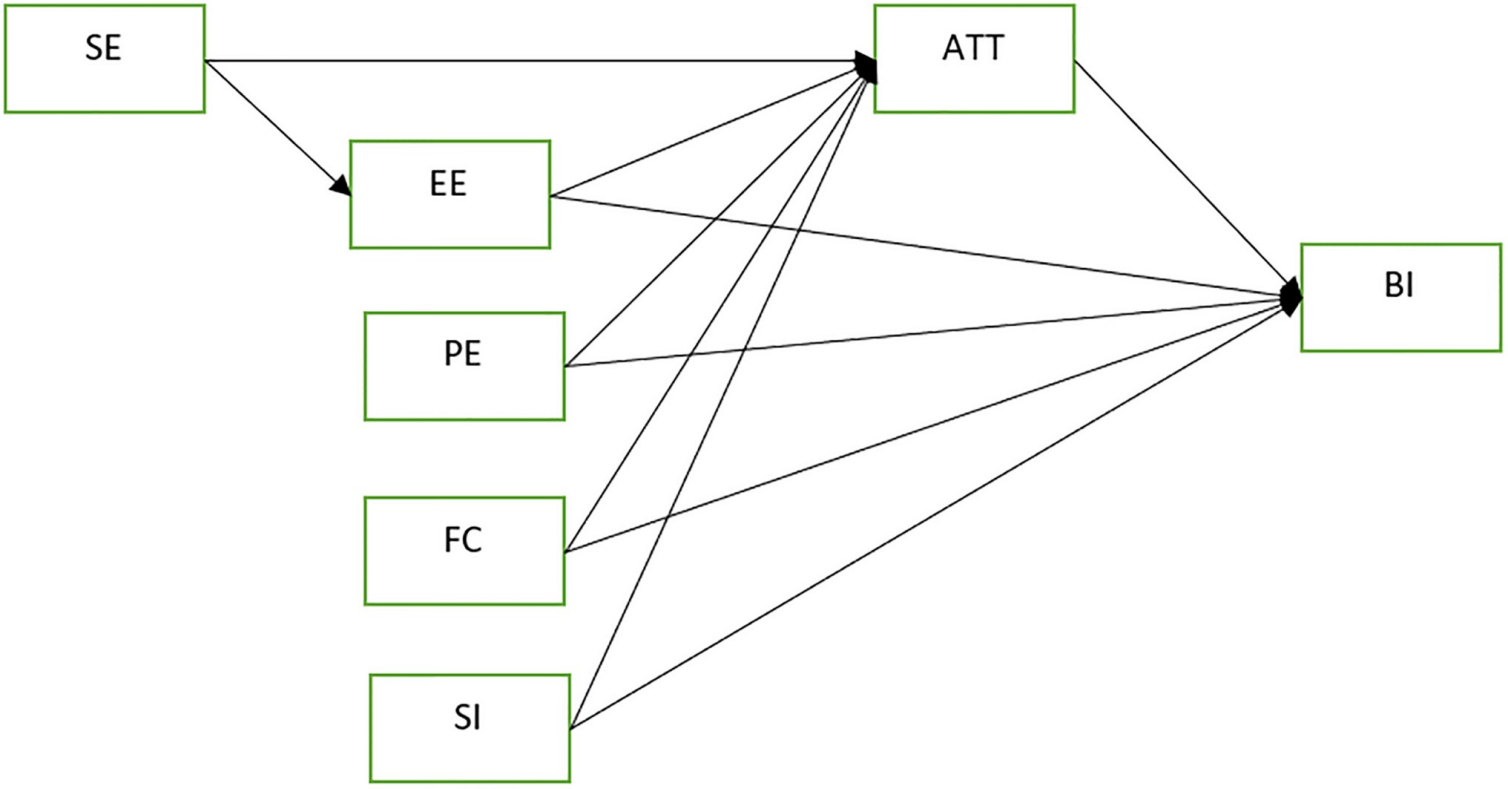
The proposed modified UTAUT model.

Technology acceptance models are usually open for modification and extension due to the evolving nature of human behavior, practice and technology and the need for contextualizing constructs to a target population. The Extended UTAUT model by Shiferaw and Mehari is by far the latest version of UTAUT model applied in resource limited settings with couple of suggested context sensitive constructs [10]. The extended UTAUT model proposed that, Intention to use technology is influenced by attitude toward the technology, performance expectancy and the level of social influence whereas actual use of technology is a function of intention to use, effort expectancy, self-efficacy and facilitating conditions [10]. Due to the unclear status of actual use of telemedicine in the setting, the construct “Actual use” was not used and the hypotheses were explained on Fig 1.

### Self-Efficacy (SE)

Self-efficacy is defined as “one’s perceived know-how and ability to utilize digital devices like computers to perform a specific task effectively” [11]. Studies have shown that user’s perceived level of self-efficacy is a critical factor in adopting digital technologies with a potential of causing frustration and anxiety [12]. Since telemedicine is an emerging technology and the fact that it requires a certain level of digital competency, the current study hypothesizes that user’s self-efficacy contributes in explaining the variance in acceptance of telemedicine in this setting. The proposed hypotheses were:

*H1*: *SE have a positive influence in EE*
*H2*: *SE have a positive influence on user’s attitude toward using telemedicine (ATT)*

### Effort Expectancy (EE)

Effort expectancy refers to “user’s perception on how easy telemedicine system is and it is steadily stated in literatures that the level of user’s perception on how easy the system is, highly affects user’s intention to use the proposed technology” [13–15]. Thus, the current study hypothesized that:

*H3*: *EE have a positive influence on user’s attitude toward using telemedicine (ATT)*
*H4*: *EE have a positive influence on user’s intention to use telemedicine system (BI)*

### Performance Expectancy (PE)

Performance expectancy is referred as “the extent in which user’s perceive that the new system could benefit in doing their job”. Studies have also indicated that user’s perception regarding the system’s benefit in their job have high influence on their attitude and intention to use the system [13, 16–18]. Thus, the current study hypothesized that:

*H5*: *PE have a positive influence on user’s attitude toward using telemedicine (ATT)*
*H6*: *PE have EE have a positive influence on user’s intention to use telemedicine system (BI)*

### Facilitating Conditions (FC)

The extent to which users perceive that there are some infrastructural and organizational facilitating conditions to use the intended systems [10]. Studies constantly indicated that both infrastructural and organizational issues are important variables to be considered in the process of adopting new information systems [19–21]. Thus, the present study hypothesized that facilitating conditions have influence on user’s attitude and intention to use the system.

*H7*: *FC have a positive influence on user’s attitude toward using telemedicine (ATT)*
*H8*: *FC have EE have a positive influence on user’s intention to use telemedicine system (BI)*

### Social Influence (SI)

Social influence is about “user’s perception on the extent to which important others like administrative staffs and colleagues support the use of the new system” [16]. Studies identified that social desirability effect and perception of important others have a direct influence on user’s attitude and intention to use the new system [21–23]. Therefore, the present study hypothesized that:

*H9*: *SI have a positive influence on user’s attitude toward using telemedicine (ATT)*
*H10*: *SI have a positive influence on user’s intention to use telemedicine system (BI)*

### Attitude (ATT)

Attitude is defined as “an intellectual and emotional entity that shows how people reason, perceive, and incline to act with regard to an event or object”. Several studies in lower-income settings identified attitude as a major significant factor that influence smooth adoption of technologies in health sector [24–26]. Thus, the present study hypothesized that:

*H11*: *ATT have a positive influence on user’s intention to use telemedicine system (BI)*

## Methods

A multi-centric online survey was conducted via social media platforms such as telegram channels, Facebook groups/pages and email during Jul 1- Sep 21, 2020. Facebook has about 13.6 million (59.72%) [27] users from the total of estimated 24.3 million data and internet users in Ethiopia [28]. Telegram is also the most preferred way of messaging platform used. Considering the pandemic’s nature of complex transmission and restrictions in transportation and other safety measures, it was reasonable to use online data collection method. The sample size was estimated by using assumptions of 50% proportion of response rate, 95% confidence interval (CI), and 5% precision with 10% non-response rate. Accordingly, a total of 423 participants were considered representative for the current survey. Healthcare providers working in COVID-19 operation centers, hospitals and other health facilities were eligible to participate in this survey. Ethical clearance was obtained from Debre Markos University ethical review board and written informed consent from the study participants were maintained.

### Measurement model assessment

The questionnaire was adopted from previously validated model in low income setting [10]. Both the constructs suggested by modified UTAUT model, Self-efficacy (SE) and Attitude (ATT) were included in the questionnaire. Overall, the questionnaire has two sections, the first section includes four (4) items regarding participants socio-demographic information, and the second section comprises the rest thirty-one (31) items aimed to assess acceptance and preference of telemedicine modalities. The questionnaire was designed using the most popular, unlimited and free online survey platform (Google forms). Before distributing the survey link, response limit to a single response from a single device, all questions were set as a required, no personal information like name, identification number, email address and digital signature were not collected to keep the respondents’ anonymity. After the Authors discussed the layout and structure of the survey, it was posted on health professional groups on Facebook, Telegram and emailed it to group contact lists of health professionals. All the required information such as consent, confidentiality and objectives of the survey were described on the first page of the survey.

Internal consistency of items was assessed using Cronbach alpha (α), composite reliability (CR) and average variance extracted (AVE) to evaluate both discriminant and convergent validity of constructs.

### Structural model assessment

Descriptive analysis was performed to summarize participant’s socio-demographic characteristics after the data was exported to SPSS Version 23. The extent of relationship among variables were evaluated by Structural equation modeling (SEM) using SPSS Amos version 23. The multidimensionality and validity of the proposed theoretical model was assessed using Confirmatory Factor Analysis (CFA). Consequently, all hypotheses from the studies was also tested. Chi-square (p-value>0.05), goodness of fit index (GFI > 0.95), adjusted goodness of fit index (AGFI > 0.95), normal fit index (NFI > 0.95), comparative fit index (CFI >0.95) and root mean square of standardized residual (RMSR < 0.05) to assess the global structural model fitness of the model. Assumptions of multivariate normality, multicollinearity, sample size appropriateness and positive definiteness were checked.

## Results

From the expected 423 responses, 319 (75.4%) participants responded to the survey questionnaire during the data collection period. The majority of participants were male (78.1%), age <30 (76.8%) and had less than five years of experience (78.1%). See Table 1 for detail.

**Table 1:** Sociodemographic characteristics of participants.

Socio-demographic characteristics	Number	Percent
**Age**		
<30	245	76.8
31–40	63	19.7
>40	11	3.4
**Sex**		
Male	249	78.1
Female	70	21.9
**Profession type**		
Doctor	194	60.8
Nurse	83	26.0
Other*	42	13.2
**Work Experience**		
<5	249	78.1
6–10	49	15.4
>10	21	6.6

*Other indicate pharmacy, midwifery, medical laboratory and health officer.

### Measurement model assessment

As presented in Table 2, the reliability and convergent validity of items and constructs were assessed using Cronbach’s alpha (Cα), composite reliability (CR) and average variance extracted (AVE). Constructs with Cα and CR value > 0.7 and AVE >0.5 were considered acceptable [29]. Convergent validity (CV) was established by evaluating CR and AVE. Accordingly, all constructs demonstrated acceptable level of reliability and validity. (See Table 2 for detail).

**Table 2:** Measurement model evaluation matrix.

Constructs	Items	SL	Cα	CR	AVE	CV
Performance Expectancy	PE1	0.79	0.91	0.91	0.73	Established
	PE2	0.90				
	PE3	0.91				
	PE4	0.80				
Self-Efficacy	SE1	0.91	0.82	0.83	0.63	Established
	SE2	0.66				
	SE3	0.79				
Effort Expectancy	EE1	0.81	0.90	0.90	0.69	Established
	EE2	0.82				
	EE3	0.87				
	EE4	0.84				
Social Influence	SI1	0.57	0.76	0.84	0.57	Established
	SI2	0.78				
	SI3	0.86				
	SI4	0.79				
Facilitating Condition	FC1	0.93	0.83	0.92	0.65	Established
	FC2	0.79				
	FC3	0.74				
	FC4	0.89				
	FC5	0.77				
	FC6	0.69				
Attitude	ATT1	0.79	0.79	0.88	0.56	Established
	ATT2	0.80				
	ATT3	0.75				
	ATT4	0.68				
	ATT5	0.83				
	ATT6	0.62				
Behavioral Intention	BI1	0.76	0.84	0.85	0.64	Established
	BI2	0.82				
	BI3	0.83				

SL = Standard loading, Cα = Cronbach alpha, CR = composite reliability, AVE = average variance extracted, CV = convergent validity.

Discriminant validity refers to the degree to which each construct measure different variables and it was assessed by comparing the squared correlation coefficients with the respective values of average variance extracted (AVE) values [30]. Discriminant validity was considered established if the AVE values of each construct is greater than the squared correlation coefficient between the constructs [30]. Consequently, Table 3 indicated that all the AVE values are greater than the squared correlations between construct which demonstrates acceptable level of discriminant validity.

**Table 3:** Discriminant validity.

Constructs correlation	Squared correlation coefficient	AVE (Left, Right)	DV
**FC<-->EE**	0.64	0.65, 0.69	Established
**SI<-->EE**	0.41	0.57, 0.69	Established
**SE<-->EE**	0.61	0.63, 0.69	Established
**PE<-->EE**	0.46	0.73, 0.69	Established
**BI<-->EE**	0.46	0.64, 0.69	Established
**ATT<-->EE**	0.39	0.56, 0.69	Established
**FC<-->SI**	0.53	0.65, 0.57	Established
**FC<-->SE**	0.42	0.65, 0.63	Established
**FC<-->PE**	0.34	0.65, 0.73	Established
**FC<-->BI**	0.47	0.65, 0.64	Established
**ATT<-->FC**	0.49	0.56, 0.65	Established
**SI<-->SE**	0.42	0.57, 0.63	Established
**SI<-->PE**	0.45	0.57, 0.73	Established
**BI<-->SI**	0.52	0.64, 0.57	Established
**ATT<-->SI**	0.50	0.56, 0.57	Established
**PE<-->SE**	0.56	0.73, 0.63	Established
**BI<-->SE**	0.52	0.64, 0.63	Established
**ATT<-->SE**	0.68	0.56, 0.63	Established
**BI<-->PE**	0.50	0.64, 0.73	Established
**ATT<-->PE**	0.49	0.56, 0.73	Established
**ATT<-->BI**	0.52	0.56, 0.64	Established

AVE = average variance extracted, DV = Discriminant validity.

### Structural model evaluation

The structural model was evaluated using the most common model fit indices and all exhibited satisfactory level of fitness. The model fit indices were Chi-square (***p***-value>.05), adjusted goodness of fit index (AGFI > 0.9), goodness of fit index (GFI > 0.9), comparative fit index (CFI >0.9), normal fit index (NFI > 0.9), and root mean square of standardized residual (RMSEA < 0.05) [31].

The structural model result confirmed the hypothesis “self-efficacy has a significant positive effect on effort expectancy” with a standardized coefficient estimate (β) of 0.76 and p-value <0.001. The result also indicated that self-efficacy, effort expectancy, performance expectancy, facilitating conditions and social influence have a significant direct effect on user’s attitude toward using telemedicine. User’s behavioral intention to use telemedicine was also influenced by effort expectancy and attitude. As indicated in Table 4, the model also ruled out that performance expectancy, facilitating conditions and social influence does not directly influence user’s intention to use telemedicine.

**Table 4:** Structural model evaluation.

Hypotheses		Standardized coefficient estimate (β)	Result
**Hypothesis 1 (H1)**	SE → EE	0.76	Supported^a^
**Hypothesis 2 (H2)**	SE → ATT	0.43	Supported^a^
**Hypothesis 3 (H3)**	EE → ATT	0.25	Supported^b^
**Hypothesis 4 (H4)**	EE → BI	0.27	Supported^b^
**Hypothesis 5 (H5)**	PE → ATT	0.70	Supported^a^
**Hypothesis 6 (H6)**	PE → BI	-0.11	Not Supported^c^
**Hypothesis 7 (H7)**	FC → ATT	0.35	Supported^b^
**Hypothesis 8 (H8)**	FC → BI	-0.18	Not supported^c^
**Hypothesis 9 (H9)**	SI → ATT	0.34	Supported^b^
**Hypothesis 10 (H10)**	SI → BI	-0.18	Not supported^c^
**Hypothesis 11 (H11)**	ATT→ BI	0.93	Supported^a^

^a^ = P-value<0.001,

^b^ = P-value<0.05,

^c^ = Insignificant.

Note. Model fit indices: χ2 = 3.93 p-value = 0.14, AGFI = 0.93, GFI = 0.99, CFI = 0.99, NFI = 0.99, RMSEA = 0.064.

The squared multiple correlations (r^2^) value indicated that 57.1% of the variance in attitude toward using telemedicine and 63.6% of the variance in behavioral intention to use telemedicine is explained by the current structural model.

### Mediation effect

As indicated in Fig 1, there are seven possible mediation paths in the model and each of them were tested for their effect and significance level. To confirm the mediation effect of mediating constructs, absolute value of computed z-scores were compared with 95% confidence level or ±1.96 and if the value of z-score is greater than the confidence level, the construct is considered as a significant mediator between constructs. To calculate z-score, unstandardized regression estimates of each path and pooled standard error of constructs in the mediation hypothesis were used [32]. Accordingly, the result showed that effort expectancy has a significant mediation effect between self-efficacy and attitude toward using telemedicine constructs and also self-efficacy and behavioral intention to use telemedicine constructs. As indicated in Table 5, the mediation effect of attitude between effort expectancy and behavioral intention, facilitating condition and behavioral intention and also social influence and behavioral intention were statistically insignificant.

**Table 5:** Mediation effect effort expectancy and attitude.

Mediation	z-score	Supported?
**SE→ATT→BI**	3.03	Yes
**SE→EE→ATT**	-3.85	Yes
**SE→EE→BI**	5.25	Yes
**EE→ATT→BI**	-0.47	No
**PE→ATT→BI**	2.39	Yes
**FC→ATT→BI**	0.69	No
**SI→ATT→BI**	0.61	No

### Telemedicine modality preferences

Telemedicine modalities are mainly categorized in to four major categories and the result from the current study used a ratting scale of one to four for each category of telemedicine modality. The most rated or preferred mode of telemedicine in this study was mobile health (mHealth) approach with 115 (36.1%) of participants rated it as their first choice. As indicated in Table 6, the second and third preferred modalities were Livestreaming (online communication) and record and send (store and forward) modalities with 30.7% and 27.3% respectively.

**Table 6:** Preference of telemedicine modalities.

Telemedicine Modalities	1^st^ Rated preference	2^nd^ Rated preference	3^rd^ Rated preference	4^th^ Rated preference
**Mobile Health (mHealth)**	**115 (36.1%)**	82 (25.7%)	78 (24.5%)	44 (13.8%)
**Livestreaming (Realtime communication)**	88 (27.6%)	**98 (30.7%)**	81 (25.4%)	52 (16.3%)
**Record and send (Store and forward)**	77 (24.1%)	73 (22.9%)	**87 (27.3%)**	82 (25.7%)
**Remote Patient monitoring (RPM)**	39 (12.2%)	66 (20.7%)	73 (22.9%)	**141 (44.2%)**

**Fig 2:**
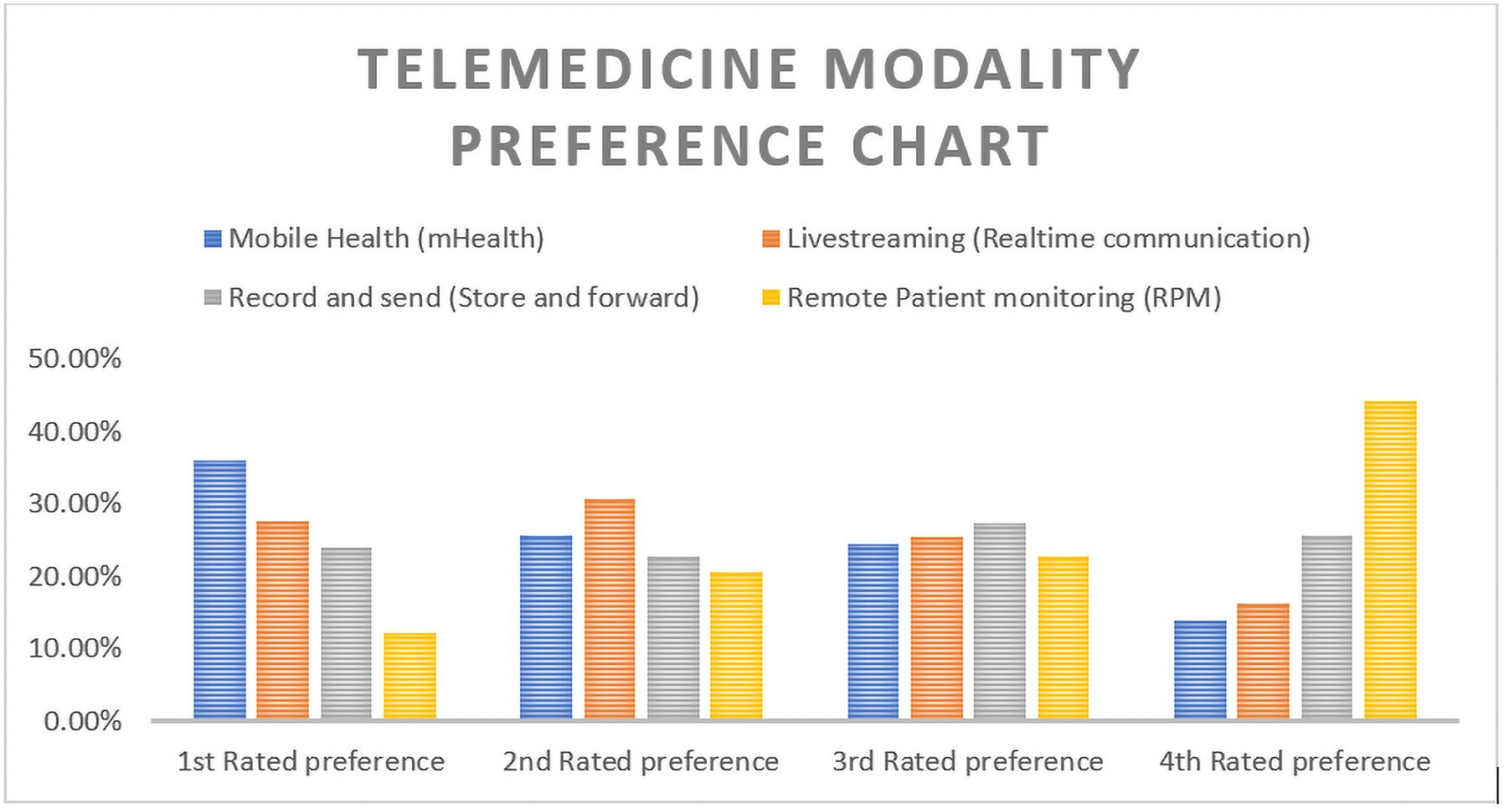
Telemedicine modality preference.

The result also showed that remote monitoring is the least preferred mode of telemedicine with 44.2% of health professionals rated as their least preferred modality. See Fig 2 for detail.

## Discussion

The result indicated that user’s acceptance of telemedicine during the COVID-19 pandemics was mainly a function of user’s perception on how easy telemedicine system is (EE) and their attitude toward using the systems (ATT). Users attitude was also significantly influenced by their perceived ability to use telemedicine systems (SE), perceived easiness of the systems (EE), perceived benefit of the system in improving their performance (PE), infrastructural and organizational facilitating conditions (FC) and the perception of important others (SI). The result also identified significant mediation effect of effort expectancy and attitude constructs in the model. The finding also depicted that mobile health (mHealth) approach was the most preferred modality of telemedicine.

The measurement model evaluation indicated that most of the constructs demonstrated an acceptable level of validity and reliability which is consistent with several studies [10, 33].

This study showed that acceptance of telemedicine system was highly influenced by users’ perception on how easy the system would be with a standardized coefficient estimate (β) of 0.27 and p-value<0.05. This implies that there is a reasonable level of improvement in understanding the dynamics of telemedicine. That means it could be as complex as conducting a surgical procedure using advanced equipment through real-time/live interaction (telesurgery) and as simple as a telephone conversation between two patients and healthcare providers. This result was in line with studies conducted in Pakistan, Nigeria and other countries that discussed the significance of user’s perceived easiness of telemedicine system in predicting overall acceptance [34–36]. Attitude was also a significant predictor of acceptance of telemedicine with β = 0.93 and p-value<0.001. Due to the nature of COVID-19 pandemics complex nature of transmission, it is reasonable to say that health professionals’ attitude toward using of telemedicine was amplified and resulted in higher intention to use telemedicine systems. The result was very consistent with other studies conducted during COVID-19 pandemics [37–39]. The finding has also shown that health professionals’ attitude toward using telemedicine was mainly influenced by self-efficacy, performance expectancy and facilitating conditions with standardized coefficient estimate of 0.43, 0.70 and 0.35 respectively with p-value <0.05. This implies that investing on improving health professionals’ knowledge and perception could result in higher level of attitude toward using telemedicine systems. The finding was in line with some studies [40, 41] and in contrast with another study [42] which highlighted that performance expectancy, effort expectancy and social influence have no significant influence on BI. The possible reason for this discrepancy could be resulted from the global urge in recommending the application of technological artifacts in health service during the COVID-19 pandemics. The risk of physical contact during the pandemics could probably be also the driving force for healthcare professionals across the world to look for more safe ways of health service delivery. The result also indicated that organizational and infrastructural facilitating conditions, the opinions of important others were also insignificant in predicting users’ acceptance of telemedicine during the pandemics. This implies that health professionals are highly motivated to apply telemedicine irrespective of their perceived knowledge, the opinions of important others and even the organizational and infrastructural preconditions. This is perhaps a unique pattern of paradigm shift that is probably resulted after the deadly pandemics. This paradigm shift could be associated with the potential threat of infection up on a physical contact between patients and health care providers. The significant mediation effect of effort expectancy and attitude also clearly asserts the indirect effect of self-efficacy and performance expectancy on predicting healthcare professionals’ acceptance of telemedicine.

Practically, preference of telemedicine modality is mainly based on sociodemographic, economic and other context sensitive variables. In this study, the most preferred modality of telemedicine was mobile health (mHealth) approach and this could be due to easy access of mobile phones and expanding telephone network. Given the infrastructural and skilled human resource limitations in low-income settings, mobile phone-based interventions are reported as effective methods in improving adherence, compliance, early detection and prevention [43, 44] In addition, the mobile phone access and penetration in low-income countries have phenomenally increased in the past decade indicating a huge potential of success in addressing larger population. Therefore, an intervention using mobile phone could probably result in better success in implementing telemedicine in resource constrained environments like Ethiopia. This finding was similar with previous studies[45, 46]. The reason behind the finding that remote monitoring modality of telemedicine was the least preferred could be due to economic limitations and other important sociodemographic variable such as educational status of patients.

### Limitation

The present study shares the limitations of online surveys. First, online surveys are completed only by persons who are computer literate and who have internet access, and probably by those who are sufficiently biased to be interested in the subject [47]. Thus, generalizations should be made carefully and further researches are required to explore and evaluate technology acceptance models in resource constrained environments.

## Conclusion

This study found that effort expectancy and attitude were significant predictors of healthcare professionals’ acceptance of telemedicine. Attitude toward using telemedicine systems was also highly influenced by performance expectancy, self-efficacy and facilitating conditions. effort expectancy and attitude were also significant mediators in predicting users’ acceptance of telemedicine. In addition, mHealth approach was the most preferred modality of telemedicine and this opens an opportunity to integrate telemedicine systems in the health system during and post pandemic health services in low-income countries.

## Supporting information

S1 Dataset (SAV)
